# Macro‐ and Nano‐Porous Ag Electrodes Enable Selective and Stable Aqueous CO_2_ Reduction

**DOI:** 10.1002/smll.202409669

**Published:** 2024-12-23

**Authors:** Behnam Nourmohammadi Khiarak, Gelson T. S. T. da Silva, Valentine Grange, Guorui Gao, Viktoria Golovanova, F. Pelayo de García de Arquer, Lucia H. Mascaro, Cao‐Thang Dinh

**Affiliations:** ^1^ Department of Chemical Engineering Queen's University Kingston ON K7L 3N6 Canada; ^2^ Interdisciplinary Laboratory of Electrochemistry and Ceramics Department of Chemistry Federal University of Sao Carlos São Carlos São Paulo 13565‐905 Brazil; ^3^ Institut National des Sciences Appliquées (I.N.S.A) de Rouen Normandie 685 Avenue de l'Université Saint‐Étienne‐du‐Rouvray 76800 France; ^4^ ICFO–Institut de Ciències Fotòniques The Barcelona Institute of Science and Technology Barcelona 08860 Spain

**Keywords:** aqueous‐fed CO_2_ electroreduction, CO electrosynthesis, CO_2_ capture, electrocatalysis, nanoporous Ag metal mesh

## Abstract

Electrochemical carbon dioxide (CO_2_) reduction from aqueous solutions offers a promising strategy to overcome flooding and salt precipitation in gas diffusion electrodes used in gas‐phase CO_2_ electrolysis. However, liquid‐phase CO_2_ electrolysis often exhibits low CO_2_ reduction rates because of limited CO_2_ availability. Here, a macroporous Ag mesh is employed and activated to achieve selective CO_2_ conversion to CO with high rates from an aqueous bicarbonate solution. It is found that activation of Ag surface using oxidation/reduction cycles produces nanoporous surfaces that favor CO_2_‐to‐CO conversion. Notably, it is found that a combination of dissolved CO_2_ in bicarbonate solution with CO_2_ generated in situ from bicarbonate ions enables increased CO_2_ availability and a CO_2_‐to‐CO conversion rate over 100 mA cm^−2^. By optimizing the oxidation/reduction cycles to fine‐tune the structure of Ag surface, CO_2_‐to‐CO conversion is reported from a bicarbonate solution with CO Faradaic efficiency of over 85% at current density of 100 mA cm^−2^, high concentration of 24.7% at outlet gas stream and stability of over 100 h with maintaining CO FE over 85% during whole reaction time.

## Graphical abstract

Aqueous‐phase systems offer a platform for electrochemical CO_2_ reduction with high CO_2_ utilization and concentrated gas products. This work explores silver Ag electrodes for CO production from aqueous‐phase CO_2_ electroreduction. Combining structural optimization and a unique “on/off” operational strategy, the system achieves a CO Faradaic efficiency above 85%, sustained over 100 h, and delivers high CO concentrations in the outlet gas stream.

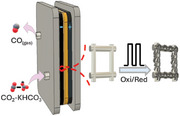

## 1 Introduction

The rising levels of atmospheric carbon dioxide (CO_2_) have prompted significant research into sustainable methods for its reduction, with electrochemical CO₂ reduction (ECR) emerging as a particularly promising approach.^[^ ^1^ ^]^ ECR could mitigate the greenhouse effect by converting CO_2_ into valuable products that are widely used today, such as carbon monoxide (CO), formic (HCOOH), methane (CH_4_), ethylene (C_2_H_4_) and ethanol (CH_3_OH), using renewable energy sources.^[^ ^2^ ^]^ Among the potential products, CO stands out due to its role in syngas production, a key intermediate for producing methanol, ammonia, and synthetic hydrocarbon fuels via the Fischer–Tropsch process.^[^ ^2^, ^3^ ^]^

High‐rate ECR systems are often based on gas‐phase CO_2_ electrolysis. In gas‐phase ECR systems, a hydrophobic gas diffusion electrode separates gas and electrolyte domains, concentrating gas reactant in a catalyst‐liquid–gas highly dynamic interface. This enables efficient mass transport and high concentrations of CO_2_ at the catalyst surface, which are essential for achieving high reaction rates and selectivity.^[^ ^4^ ^]^

Current gas‐phase ECR systems face challenges related to long‐term operation due to the low stability of gas diffusion electrodes, mainly caused by loss of hydrophobicity, electrolyte flooding, and salt formation.^[^ ^5^ ^]^ During ECR, the aqueous electrolyte penetrates through the pores of the GDE, thus blocking the diffusion of CO_2_ to the catalyst layer, and lowering CO_2_ reduction current. Inorganic salt formation inside the GDE or on the catalyst layer can also block CO_2_ diffusion and active sites, precluding ECR.^[^ ^5^, ^6^ ^]^ To date, the most studied systems for gas‐phase CO_2_ reduction are based on anion‐exchange membrane (AEM).^[^ ^7^ ^]^ However, in this configuration, CO_2_ crossover to the anode side requires additional energy cost for CO_2_/O_2_ separation in the anode.^[^ ^8^ ^]^

Recently, there has been growing interest in developing electrolyzers capable of converting aqueous CO_2_ streams—solutions containing dissolved CO_2_–into valuable commodity chemicals.^[^ ^8^, ^9^ ^]^ This configuration would eliminate the need for hydrophobic gas diffusion layers, in principle overcoming challenges associated with flooding and salt formation in conventional gas‐phase ECR. In addition, high gas product concentration can be achieved with aqueous phase CO_2_ reduction.^[^ ^8^, ^10^ ^]^ CO_2_ sources for aqueous phase systems can be provided by two methods. The first one involves dissolving CO_2_ from aqueous solution. The second source involves CO_2_ generated in situ from a bicarbonate solution via local protons during electrocatalysis processes.^[^ ^8^, ^10^ ^]^ One of the biggest challenges with aqueous CO_2_ reduction is the low CO_2_ reduction rate due to the limitation of CO_2_ availability.^[^ ^8^, ^10^ ^]^

To achieve a high CO_2_ reduction rate in aqueous systems, electrode structures have been optimized for either dissolved CO_2_ or bicarbonate systems.^[^ ^11^ ^]^ With dissolved CO_2_, highly porous electrodes, including metal mesh and foam with large pores, which facilitate electrolyte transport, have been frequently used.^[^ ^9^, ^10^ ^]^ In the case of bicarbonate sources, layers of metal particles coated on a porous substrate, such as carbon papers, have been explored.^[^ ^10^, ^11^, ^12^ ^]^ To date, electrodes that effectively combine the two sources of carbon dioxide for CO production from an aqueous solution remain underexplored.

In this work, we present a strategy to increase local dissolved bicarbonate‐derived CO_2_ based on macro‐nano‐porous Ag‐based electrodes. Our Ag electrodes comprise two porous features: i) a very large pore (macroporous) from the mesh structure which enhances the mass transport for bicarbonate and dissolved CO_2_ for high CO_2_ availability; and ii) a nanoporous structure as a result of cyclic voltammetry (CV) activations that enables high local pH for selective CO_2_ reduction to CO. As a result, the designed electrode exhibits 85% CO faradaic efficiency (FE) at current densities between 50 and 100 mA cm^−^^2^ while without CV activation of Ag the CO FE was less than 2%. And a CO partial current density exceeding 100 mA cm^−^^2^ at the current densities of 150–200 mA cm^−2^ was achieved. The system is also stable, maintaining high CO FE (>85%) for at least 100 hour (h) at a current density of 100 mA cm^−^^2^. Additionally, the system delivers a high CO concentration of up to 24.7% in the gas outlet stream.

## 2 Results and Discussion

### 2.1 In Situ Reconstruction of Selective Catalyst

**Figure 1:**
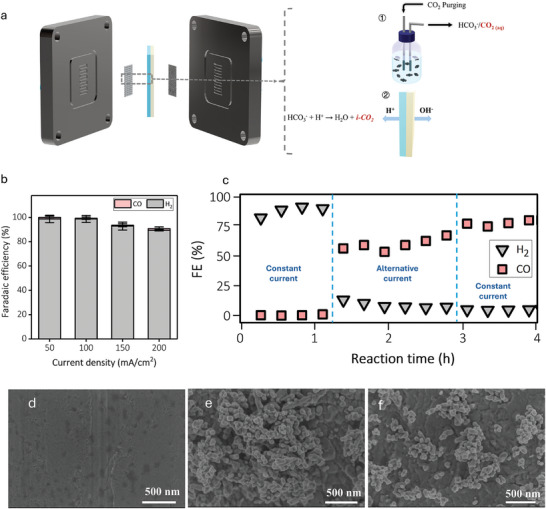
a) A schematic demonstration of bicarbonate electrolyzer system and sources of CO_2_ for subsequent ECR; 1) dissolved CO_2_ source, 2) in situ generated CO_2_ from bicarbonate ions reacting with H^+^, b) FEs versus current densities of bare Ag mesh, c) CO and H_2_ FE overtime under different stages of the reaction, and d–f) SEM images of Ag before, after pulse electrolysis, and after switching to constant current.

We employed an aqueous system using bicarbonate electrolyte‐saturated CO_2_ as feedstock. This system enables high‐rate CO_2_ conversion since both dissolved CO_2_ and CO_2_ generated from bicarbonate are used as carbon sources (**Figure** **1**a).^[^ ^9^ ^]^ The bicarbonate electrolyzer features a bipolar membrane (BPM) sandwiched between a porous Ni foam anode and an Ag mesh cathode (Figure 1a). During CO_2_ reduction, in situ CO_2_ is generated from the reaction between bicarbonate ions and protons from BPM (Figure 1a). Both dissolved CO_2_ and in situ CO_2_ can be converted to desired products in this electrochemical configuration. In our system, we utilized an Ag mesh with a 100‐micron pore size as the cathode (Figure 1a), leveraging the mesh structure's well‐established efficacy for CO_2_ conversion.^[^ ^9^ ^]^ The macropores enhance the diffusion of bicarbonate and dissolved CO_2_ toward the catalyst surface, improving contact and reaction efficiency.

Electrochemical CO_2_ conversion was performed at a fixed current density in the range of 50–200 mA cm^−2^ (Figure 1b). H_2_ was found to be the dominant product with a Faradaic efficiency (FE) of over 90% at all tested currents with bare Ag electrodes. Only a trace amount of CO with FE below 2% was obtained (Figure 1b). To suppress H_2_ and enhance CO production, we employed pulse electrolysis in which the currents were alternated between a positive and a negative value. This approach has been successfully employed for CO_2_ conversion to methane on a Cu catalyst.^[^ ^9^ ^]^

To employ pulse electrolysis, we first performed a reaction at a fixed current density of 100 mA cm^−2^. As expected, H_2_ was the main product (Figure 1c). Then, the program was switched to alternating currents between 100 mA cm^−2^ (reduction) for 55 s and +1.5 mA cm^−2^ (oxidation) for 5 s, we observed a significant increase in CO FE while H_2_ was suppressed. A constant CO FE of ≈60% was maintained during one hour of reaction using this alternating current program (Figure 1c). To check if pulse electrolysis was required to maintain high CO_2_ reduction selectivity as we observed previously on the Cu catalyst,^[^ ^9^ ^]^ we switched the program back to a fixed current density of 100 mA cm^−2^. To our surprise, CO FE remained high at over 75% throughout the fixed current operation (Figure 1c).

We reason that the surface of Ag is reconstructed during alternating current operation and becomes selective for CO_2_ reduction to CO. Once the surface is reconstructed, it becomes selective and stable for CO_2_ reduction. To study the change of Ag catalyst during fixed and alternating current operation, we characterized the catalysts after each step, i.e., i) after fixed current at 100 mA cm^−2^ (Figure 1d), ii) after fixed current and alternating current (Figure 1e), and iii) after fixed current, alternating current, and fixed current (Figure 1f), using Scanning Electron Microscopy (SEM). The surface of Ag was found to be unchanged after the first fixed current operation (Figure 1d; Figure S1a,b, Supporting Information). After a fixed current and alternating current, the Ag surface becomes textured (Figure 1e; Figure S1c,d, Supporting Information). The Ag surface remains textured after the last fixed current step (Figure 1f; Figure S1e,f, Supporting Information). Our results show that Ag mesh with a textured surface can be selective for CO_2_ reduction to CO in an aqueous solution‐fed system. Similar effects have been observed on Ag catalysts using gas‐phase CO_2_ reduction in flow‐cells or aqueous CO_2_ reduction in H‐cells.^[^ ^13^ ^]^

### 2.2 Tuning Surface Reconstruction

**Figure 2:**
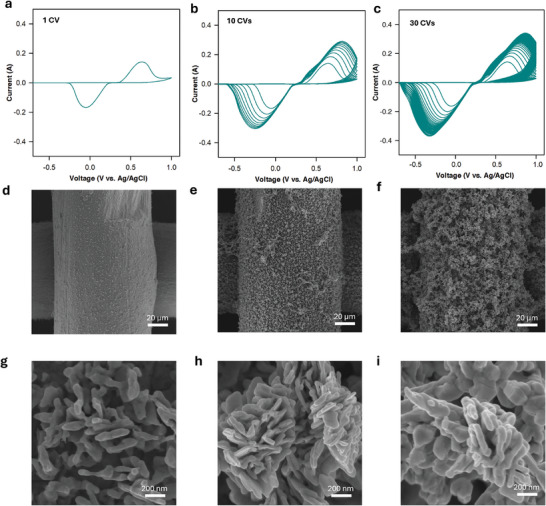
Characterization of carbonate‐derived Ag catalyst. a–c) A representative CV curve for the oxidation–reduction of Ag catalyst to change the surface at different CV cycles, d–f) low magnification SEM images for different CV‐modified Ag mesh samples with 1, 10, 30 cycles, and g–i) high magnification SEM images for different samples.

Inspired by our findings with pulse electrolysis, we sought to use a different approach to control the activation of the Ag surface. To this end, we employed CV measurement to tune the surface structure of Ag. Reconstruction of the Ag surface was performed using repeated CV within the potential range of +1 to −1 V (vs Ag/AgCl) in a 1 m KHCO_3_ electrolyte. High concentration of KHCO_3_ (1 m) was employed for the pretreatment of the Ag mesh to ensure high conductivity of the solution, which is critical for effective CV pretreatment at high currents. The number of CVs varied from 1 to 30 to tune the Ag surface (**Figure** **2**a–c).

The CV curve shows a dominant oxidation peak of Ag at +0.8 V versus Ag/AgCl and a reduction peak of Ag^+^ at −0.15 V versus Ag/AgCl (Figure 2a). Increasing the number of oxidation–reduction cycles leads to the shifting of both the oxidation and reduction peaks, along with an increase in their intensity (Figure 2a–c). To study the change in surface morphology, we performed SEM analysis of the samples after different numbers of CV cycles (Figure 2d–i). We observed a progressive development of surface roughness and porosity with increasing CV cycles. After 1 cycle (Figure 2d,g), the surface begins to show signs of restructuring. With 10 cycles (Figure 2e,h), the surface becomes more intricate, forming noticeable porous structures. At 30 cycles (Figure 2f,i), the surface exhibits a highly porous and rough morphology, indicating extensive reformation. The high magnification SEM images (Figure 2g–i) provide detailed views of these changes, where initial roughness and small features are visible after 1 cycle (Figure 2g), well‐developed nanostructures appear after 10 cycles (Figure 2h), and densely packed, highly textured nanostructures are prominent after 30 cycles (Figure 2i).

The electrochemical surface area (ECSA) measurements of the Ag mesh modified using different numbers of CVs show significant enhancement in the surface area of Ag mesh catalysts following CV activation (Figure S2a–e, Supporting Information). The double layer capacitance (C_dl_) of one CV activated‐Ag (Ac‐Ag) is 2.67 times that of bare Ag's C_dl_ (Table S1, Supporting Information). The C_dl_ values of Ag mesh increased 25.33 and 106.6 times compared to bare Ag mesh after 10 and 30 CV cycles, respectively.

**Figure 3:**
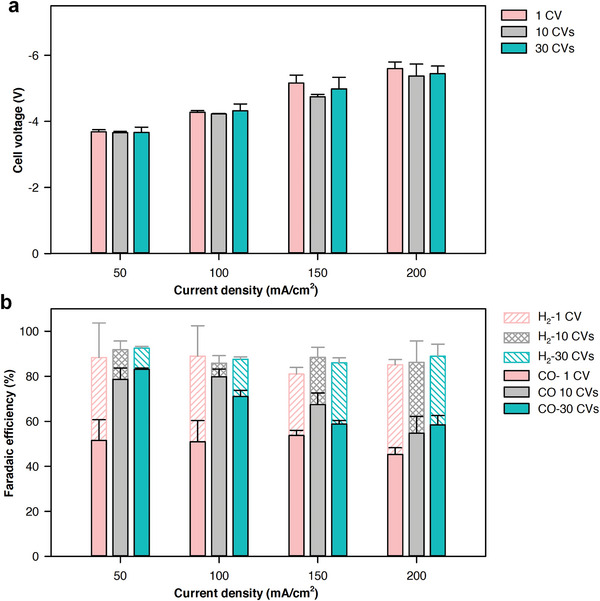
CV pre‐treatment effect. a) The cell voltage at different current densities for all three samples, and b) The ECR performance of the Ag mesh samples modified with different CV cycles: 1, 10, and 30 cycles, the scan rate for all CV cycles were the same at 50 mV s^−1^ in 1 m KHCO_3_ catholyte.

To characterize the oxidation states of Ag during CV activation, we performed X‐ray photoelectron spectroscopy (XPS) analysis for the Ag mesh sample at three different stages, including before CV treatment (bare Ag mesh), oxidized Ag (R/O, stopped after oxidation) and reduced Ag (R/R, stopped the CV after reduction) (Figure S3a, Supporting Information). The survey XPS spectrum shows the difference in surface composition of samples at different stages (Figure S3b, Supporting Information). Bare Ag and reduced Ag samples show mainly Ag with very small amounts of oxygen. In contrast, a significant amount of oxygen is presented on oxidized Ag. The high‐resolution spectra for the bare Ag and reduced Ag (R/R) primarily show well‐defined peaks at 368.28 and 374.28 eV (Figure S3c, Supporting Information), which are attributed to metallic silver. These peaks shift to lower binding energy values of 367.95 and 373.98 eV, which can be attributed to Ag^+^ in silver carbonate.^[^ ^14^ ^]^ The presence of two peaks at 284.7 and 288.83 eV in C1s spectrum of oxidized Ag samples (**Figure** **3**d) could originate from carbonate, further confirming the formation of silver carbonate after the oxidation cycle.

### 2.3 Electrochemical CO_2_ Performance

The CO_2_ reduction performance of Ac‐Ag samples was evaluated using a 0.3 m KHCO_3_ solution at current densities ranging from 50 to 200 mA cm^−2^. Activating Ag with CV did not significantly influence the cell voltage (Figure 3a). The cell voltages of all samples were in the range of 3.8–5.8 V at current density from 50 to 200 mA cm^−2^. Compared to the bare Ag mesh (Figure 1b), all Ac‐Ag samples showed much higher CO selectivity. The Ag sample modified with one CV cycle shows a maximum CO FE of 55–60% in the current density range of 100–150 mA cm^−2^ (Figure 3b). The 10‐CV sample exhibits high CO FEs of 80–83% at a current density below 100 mA cm^−2^ (Figure 3b). The CO FE decreases to 70 and ≈55% at current densities of 150 and 200 mA cm^−2^, leading to a maximum CO partial current density of ≈110 mA cm^−2^. Further increasing the number of cycles did not lead to additional improvements, with CO FE stabilizing in the range of 75–80%, similar to that of the sample modified with 10 cycles. We reason that this plateau in performance points to the system being CO_2_‐limited rather than limited by the catalytic properties of the Ag surface. Therefore, while both 10‐CV and 30‐CV treatments yield similar outcomes, the data underscore the importance of optimizing CO_2_ availability to further improve CO_2_ electroreduction efficiency.

Previous studies on porous Ag catalysts for CO_2_ reduction have pointed out two key factors that affect the catalytic activity of catalysts: i) the local pH changes due to the increased surface area and porous structure and ii) an electronic reconfiguration and active crystalline facet exposure resulting from the altered surface morphology.^[^ ^13a^ ^]^ The local pH increment, induced by the high current density and enhanced surface area, suppresses the hydrogen evolution reaction, thereby increasing CO selectivity. The electronic reconfiguration and exposure of new crystalline facets due to surface morphology changes alter the binding energy of CO_2_ electroreduction intermediates, reducing the activation energy barrier for the reduction of CO_2_ to CO, which further enhances CO selectivity.^[^ ^13^, ^15^ ^]^ We believe that these two factors are also crucial in our aqueous phase CO_2_ conversion system using porous Ag meshes.

### 2.4 Effect of Catholyte Concentration

**Figure 4:**
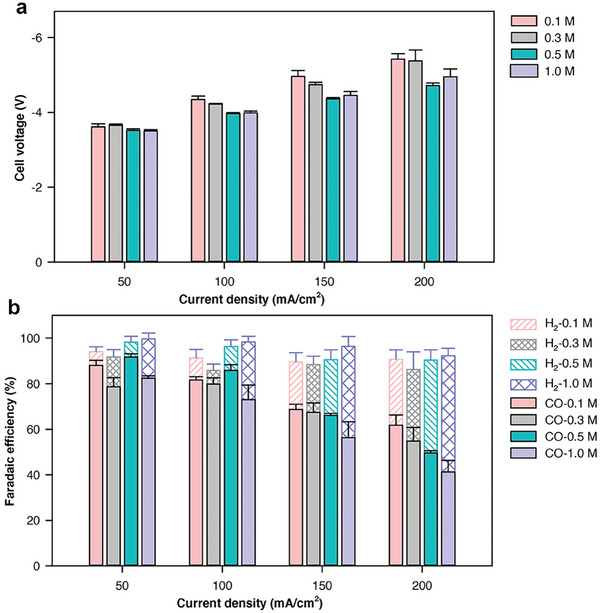
Catholyte concentration effect. a) The cell voltage versus current density for different bicarbonate concentrations, and b) effect of bicarbonate concentration on CO FE.

We investigated the effect of KHCO_3_ concentration in the catholyte on the ECR performance because it can significantly impact CO_2_ solubility, the generation of in situ CO_2_, and the local pH at the catalyst surface.^[^ ^16^ ^]^ In general, electrolytes with lower KHCO_3_ concentrations exhibit higher CO_2_ solubility and result in a higher local pH at the catalyst surface during ECR due to their reduced buffering capacity. Conversely, higher KHCO_3_ concentrations increase the availability of HCO_3_⁻ ions at the membrane surface, enhancing the in situ generation of CO_2_. At a low current density of 50 mA cm^−2^, the electrolyte concentration has a negligible effect on the overall cell voltage (**Figure** **4**a). As the current density increases, the cell voltage rises more significantly for lower KHCO_3_ concentrations which could be attributed to the lower ion conductivity of the catholyte (Figure 4a).

There is no clear trend on the effect of catholyte concentration on CO FE at low current densities of 50 and 100 mA cm^−2^. KHCO_3_ with a concentration of 0.5 m exhibits the highest CO FE in that current range. However, we observe a clear trend in CO FE at the high current densities, where CO FEs are lower with higher KHCO_3_ concentrations. Our results suggest that dissolved CO_2_ and high local pH on the surface of the catalysts, enabled by catholyte with low concentrations, are critical for high CO FEs. They also suggest that a high concentration of bicarbonate is not needed for in situ CO_2_ generation in our system.

### 2.5 CO_2_ Source Validation

**Figure 5:**
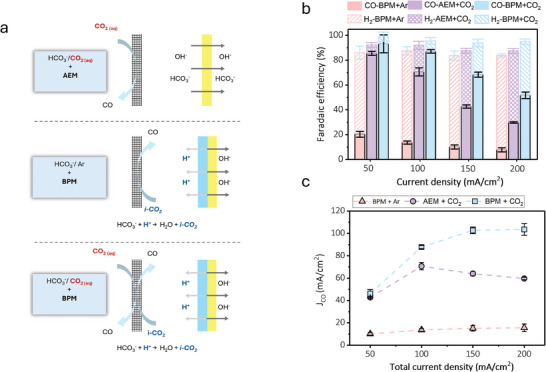
Effect of CO_2_ sources. a) A schematic demonstration of CO_2_ sources in different configurations of the cell, b) Gas product distribution at different current densities of activated Ag mesh (10 cycles of CV) conducted using different cell configurations: an AEM and CO_2_‐saturated 0.3 M KHCO_3_ electrolyte, BPM with argon‐saturated 0.3 M KHCO_3_ electrolyte, and BPM with CO_2_‐saturated 0.3 m KHCO_3_ catholyte, and c) CO partial current density at different applied current density under different cell CO_2_ source availability.

As demonstrated in previous studies,^[^ ^9^ ^]^ aqueous solution‐fed systems for ECR have two sources of CO_2_: dissolved CO_2_ in the electrolyte and CO_2_ generated in situ from protons reacting with bicarbonate. To elucidate the role of each source and their contribution to ECR, we performed a series of controlled experiments. First, we performed ECR with an AEM. In this setup, the AEM blocks positively charged ions (*i.e*., H^+^) transfer; therefore, the only source of CO_2_ would be the dissolved CO_2_ in the electrolyte (**Figure** **5**a). In this cell configuration, dissolved CO_2_ enables CO selectivity of up to 80% at the lower current density of 50 mA cm^−2^, while as the current density increases, CO FE decreases (Figure 5b), reaching 30% at the current density of 200 mA cm^−2^. With AEM and dissolved CO_2_, a maximum CO partial current density of 70.6 mA cm^−2^ was achieved (Figure 5c).

To explore the role of the in situ CO_2_ generation effect, we performed the ECR test under argon‐saturated bicarbonate catholyte and a BPM (Figure 5a). In this setup, the contribution of dissolved CO_2_ is limited to the equilibrium dissolved CO_2_ concentration of the electrolyte under the argon environment. We found that the CO selectivity under in situ‐generated CO_2_ is relatively low. The highest recorded CO FE was 20.3%, achieved at 50 mA cm^−2^ (Figure 5b). In this configuration, a low CO partial current density of 15.6 mA cm^−2^ was obtained (Figure 5c), which means that the availability of CO_2_ is significantly low and requires extra CO_2_.

These results confirm that the presence of dissolved CO_2_ is crucial for high CO FE, and both CO_2_ sources are required for high CO selectivity at high current densities, which can be obtained with CO_2_‐saturated KHCO_3_ and BPM structure (Figure 5a,b). We observed that the cumulative CO FE from single CO_2_ sources is ≈10% lower than that of the system with combined dissolved CO_2_ and in situ CO_2_ at high current densities of 150 and 200 mA cm^−2^ (Figure 5b). A similar trend was observed with CO partial current density, in which CO partial current in the system with combined CO_2_ source is much higher than the sum of CO partial currents in sole CO_2_ source systems (Figure 5c). This result suggests a synergistic effect between these two CO_2_ sources. This synergy likely optimizes the local CO_2_ concentration at the catalyst surface by increasing CO_2_ mass transport, thereby improving the selectivity and efficiency of CO production. While the contribution of in situ CO_2_ to total CO current is not significant, we believe it indirectly enhances CO_2_ availability by facilitating mass transport within Ag electrodes. During CO_2_ reduction, the reaction of bicarbonate ions and protons from the BPM forms CO_2_ bubbles on the BPM surface^[^ ^9^, ^17^ ^]^ which diffuse through the Ag electrode layer. This bubble diffusion facilitates the mass transport of dissolved CO_2_‐containing electrolytes to the Ag surface, increasing CO_2_ reduction currents.

### 2.6 Product Concentration and Catalyst Stability

**Figure 6:**
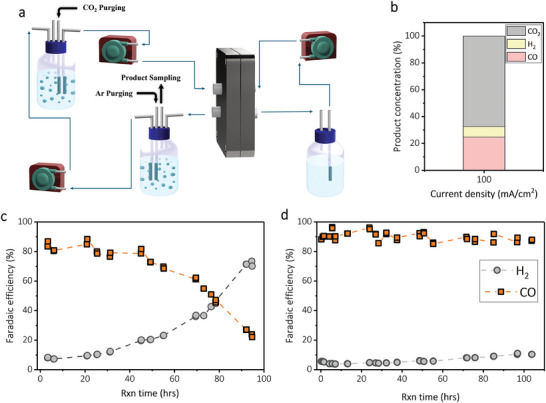
Product concentration and catalyst stability. a) Schematic illustration of the experimental setup for analyzing product concentration. b) H_2_, CO, and CO_2_ concentrations in the outlet gas stream at a fixed current density of 100 mA cm^−2^, c) Ag catalyst stability under constant 100 mA cm^−2^ operation, (d) long‐term stability with on/off under 5 s “off” regime and 55 s “on” regime at 100 mA cm^−2^.

We analyzed the product concentrations at the gas stream outlet using a custom‐designed system. In this setup, the gas product is separated from the CO_2_ bubbling solution. Both the gas and liquid electrolyte pass through a separator, where the gas products are collected for analysis, and the liquid electrolyte is recycled back to the CO_2_‐saturated solution (**Figure** **6**a). The primary gas components are CO, H_2_, and CO_2_. At a current density of 100 mA cm^−2^, a high CO concentration of 24.7% was achieved (Figure 6b). The excess CO_2_ observed in the gas stream may come from unreacted in situ and dissolved CO_2_. In principle, the number of in situ CO_2_ molecules is equal to the number of hydroxide ions (OH^−^) produced during the electrolysis reaction on the cathode surface. We reason that an efficiently designed gas/liquid contactor that facilitates the back reaction between in situ CO_2_ and hydroxide ions could enhance CO_2_ conversion to bicarbonate and reduce the amount of CO_2_ in the gas product stream.

We then examined the stability of the 10 cycles of Ac‐Ag samples under continuous reaction conditions at a current density of 100 mA cm^−2^ in a 0.3 m KHCO_3_ catholyte. The Ac‐Ag catalyst initially maintained a CO FE of ≈80% for the first 45 h (Figure 6c). However, the CO FE dropped rapidly to less than 50% within 75 h of continuous operation and reached 20% within 100 h of operation. Moreover, the H_2_ FE constantly increased during the reaction (Figure 6c). The cell voltage gradually increased throughout the test (Figure S4a, Supporting Information). It indicated the rising resistance within the system, which could be caused by impurity buildup on the Ag catalyst surface, changes in electrolyte composition, or BPM delamination.

The SEM images, taken after 100 h of reaction, showed significant reconstruction in the surface morphology in comparison to the sample before the reaction (Figures S5a and S4b, Supporting Information). The EDS analysis also revealed the presence of silicon (Si) deposition on the surface (Figure S4c, Supporting Information), which could be sourced from silicone tubes used for electrolyte pumps. Therefore, we hypothesize that the increase in H_2_ FE over time, coupled with the decline in CO FE, is likely due to the deposition of impurities within the inner porous sites of the catalyst. It suggests that maintaining the cleanliness of the catalyst surface and preventing impurity deposition could be a way to sustain high CO FE over prolonged operation periods.

To improve the system stability, we implemented a regeneration strategy using an electrolysis method with alternating turning on the applied current source which is defined as the “on” segments, and turning off the applied current source which is defined as “off” segments. A 55 s “on” segment, applying a reduction current density of100 mA cm^−2^, then an “off” segment for 5 s was applied. These cycles repeated over the whole reaction time. We hypothesized that the “off” regimes would help to refresh the catalyst surface. The intermittent pauses in operation were expected to facilitate the leaching of small amounts of deposited impurities back into the solution. Through this approach, we have observed a significantly increased operating lifetime of the Ag catalyst, maintaining a CO FE of ≥85% for at least 100 h of electrolysis in a 0.3 m KHCO_3_ medium at a current density of 100 mA cm^−2^ (Figure 6d). The H_2_ FE remained below 10% during the whole reaction time (Figure 6d). Unlike the continuous operation test, the cell voltage remains relatively stable over the entire duration of the test (Figure S4c, Supporting Information). The “off” regimes likely allow for the relaxation and recovery of the electrode surface, potentially reducing the buildup of impurities and mitigating degradation.

From the SEM analysis after 100 h of operation, we also observed surface reconstruction which is similar to our observation under constant current stability test (Figures S5b and S4d, Supporting Information). It is worth noting that surface reconstruction of Ag during CO_2_ electroreduction mainly influences the total activity (current density) due to changes in surface area or morphology but does not significantly affect the FE for CO. This robustness arises from Ag's intrinsic electronic properties, which favor CO production under a wide range of structural configurations. Also, the EDS analysis showed a negligible Si peak intensity as a deposited impurity (Figure S4f, Supporting Information). We believe that during “off” segments, most of the deposited impurities are leached back to the solution (Figure S4f, Supporting Information). This result supports our hypothesis that the impurity deposition and other potential chemical alterations of the Ag surface are the major contributors to the instability of the catalyst under constant current operation.

We also conducted X‐ray diffraction (XRD) analysis for the catalysts after extended stability tests. As shown in Figure S6 (Supporting Information), the XRD patterns of the Ac‐Ag mesh catalyst before and after the stability tests (both constant current and on/off cycles) reveal no significant changes. The consistent diffraction peaks indicate that the crystalline structure of the catalyst remains unchanged throughout the testing period. This stability in the crystalline structure suggests that the catalyst's integrity and robustness are maintained, even under prolonged electrochemical operation.

## 3 Conclusion

In summary, we developed macro‐nano‐porous Ag electrodes for the selective and stable electroreduction of CO_2_‐bicarbonate to CO at high current densities. We found that the porous structure of Ag is critical for selective CO_2_ reduction and mass transport in aqueous CO_2_ conversion system. By optimizing the surface of porous Ag mesh, we demonstrated CO_2_‐to‐CO conversion with a CO partial current density of over 100 mA cm^−2^ in an aqueous‐fed system. By combining electrode optimization with an on/off operating strategy, we demonstrated CO_2_‐bicarbonate‐to‐CO conversion with a CO FE above 85% for over 100 h. The catalyst also delivered a high CO product concentration of 24.7% in the outlet gas stream.

## 4 Experimental Section

### Materials

The chemicals used were potassium bicarbonate (KHCO_3_, 99.5–101%, Sigma–Aldrich), potassium hydroxide (KOH, >85%, Fisher chemical), silver mesh (Ag‐mesh (100 pores per inch (ppi)). All reagents were used as received, without further purification. High pure water (18.2 MΩ cm) was used throughout the experiments. The used AEM (Sustainion Anion Exchange Membrane) and BPM (fumasep FBM single film Bipolar Membrane) were modified according to the manufacturer's guidelines.

### Synthesis

The activation of the Ag‐mesh was conducted by applying cyclic voltammetry (CV) with a scan rate of 50 mV s^−1^ between +1.0 and −1.0 V versus Ag/AgCl with different numbers of cycles (1, 10, and 30 CV). These procedures were carried out in a two‐chamber electrochemical cell, known as H‐cell, separated by a BPM, where the cathode side contained an aqueous solution of KHCO_3_ (1 m) and the anode KOH (1 m). Activated Ag‐mesh (1 × 2 cm) were used as the working electrodes, while a platinum mesh and an Ag/AgCl (3 m KCl) electrode were used as the counter electrode and reference electrode, respectively.

### Electrochemical CO_2_ Tests

ECR was carried out in the Membrane Electrode Assembly (MEA) cell, which consisted of a titanium plate cathode (anode) with an etched serpentine‐shaped flow channel (geometric area of 1 × 2 cm). In this setup, the activated Ag mesh was the cathode, while the anode was a nickel foam. To ensure no leakage or short‐circuits between the metal plates, two PTFE gaskets (0.03″ each) were placed between them with a 1 × 2 cm central cut‐out, effectively enclosing the ion exchange membrane used (AEM or BPM). Both the catholyte (KHCO_3_, 250 mL) and the anolyte (1 m KOH, 200 mL) were circulated through the electrochemical cell using peristaltic pumps. Before the ECR experiments, CO_2_ gas (99.99999%, Praxair) was continuously bubbled into the catholytes for over 3 h to saturate them and was also maintained throughout the experiment to keep the dissolved CO_2_ concentration fixed. On the other hand, experiments were also conducted using argon (Ar) (Praxair, 99.99999%) as the carrier gas to determine the portion of bicarbonate directly reacted.

During the electrolysis, the catholyte reservoir was connected in line with gas chromatography (GC, PerkinElmer Clarus 590) to qualify and quantify the gas products. This equipment was equipped with two packed columns, a molsieve 5A packed column (1.8 m × 1/8 in × 2.1 mm) (Supelco), and a carboxen 1000 packed column (1.5 m × 1/8 in × 2.1 mm) (Supelco) connected in parallel to the thermal conductive detector (TCD) and flame ionization detector (FID), respectively. The quantification of gas products and Faradaic Efficiency was calculated using a calibration curve of standard gaseous mixtures.

From the correlation of the peak area found on the GC with the values adjusted by the calibration curve, the number of products in moles (x) was determined. With this information, combined with Faraday's constant (F, given by s⋅A⋅mol^−1^), the number of electrons (n) needed to convert CO_2_ into CO (2e^−^) and the parasitic hydrogen evolution reaction (HER) (2e^−^), and the charge generated during the reaction (C, given by the applied current in ampere, multiplied by the reaction time in second), the faradaic efficiency could calculated as described below:

(1) $$FE\left(\%\right)=\frac{n.x\left(mol\right)\cdot F\left(s.A.mol^{-1}\right)}{C\left(A.s\right)}\times 100$$

### Characterization

All the electrochemical tests were performed using a potentiostat Metrohm Autolab. The analysis of the diffraction pattern of the Ag‐mesh was conducted using a Panalytical‐Empyrean X‐ray diffractometer equipped with Cu‐Kα radiation. The scan range extended from 2θ = 30°–80°, employing a continuous scan mode characterized by a step width of 0.02° and a scan speed of 2° min^−1^. X‐ray photoelectron spectroscopy (XPS) analysis was performed using a K‐Alpha instrument (Thermo Scientific) with an Al K‐alpha X‐ray source (1486.6 eV) to study the surface elemental composition and chemical states. Survey spectra were acquired with a pass energy of 200 eV, an energy step size of 1.0 eV, and 20 scans, while high‐resolution spectra were collected with a pass energy of 50 eV, an energy step size of 0.10 eV, and 10 scans. The spot size was 300 µm for all measurements, ensuring detailed surface characterization and accurate analysis of the material's chemical properties. The morphologies of the Ag‐mesh were carried out through field‐emission scanning electron microscopy (FEI‐MLA Quanta 650 FEG‐ESEM) at an accelerating voltage of 20 kV, operating with a backscattered electrons (BSE) detector. The electrochemical surface area (ECSA) of the pristine and activated Ag mesh using cyclic voltammetry (CV) was calculated by measuring the double‐layer capacitance (C_dl_). For this, electrochemical tests were carried out in a usual three‐electrode cell in 0.3 m KHCO_3_ over a potential window of 200 mV (from 0 to 0.2 V vs Ag/AgCl) at different scan rates (10, 20, 30, 40, 50, 75, and 100 mV s^−1^). This window was selected to encompass the absence of a faradaic process, an essential condition for measuring C_dl_. From the data collected, the double‐layer charge current was plotted against the scan rate (Figure S2e, Supporting Information), and the slope of the linear regression shows the double‐layer capacitance.

## Conflict of Interest

The authors declare no conflict of interest.

## Author Contributions

C.T.D. supervised the project. B.N.K. and C.T.D. designed all the experiments. B.N.K, V.G, and G.T.S.T.S. conducted experiments and data processing. V.G, G.G., and F.P.G.A. performed SEM analysis. G.T.S.T.S. and L.H.M. performed XPS analysis. All authors contributed to manuscript writing and revising.

## Supporting information



Supporting Information

## Data Availability

The data that support the findings of this study are available from the corresponding author upon reasonable request.
