# Supplementary material for: Macro‐ and Nano‐Porous Ag Electrodes Enable Selective and Stable Aqueous CO2 Reduction
Source: Small. 2024 Dec 23;21(8):2409669. doi: 10.1002/smll.202409669 (PMC11855228; doi:10.1002/smll.202409669)
Supplement: Supplementary file 1 — Supporting Information [file SMLL-21-2409669-s001.docx]

**Macro- Nano-Porous Ag electrodes enable selective and stable aqueous CO_2_ reduction**

Behnam Nourmohammadi Khiarak^1^, Gelson T. S. T. da Silva^1,2^, Valentine Grange^1,3^, Guorui Gao^1^, Viktoria Golovanova^4^, F. Pelayo Garcia de Arquer^4^, Lucia H. Mascaro^2^ and Cao-Thang Dinh^1,*^

^1^Department of Chemical Engineering, Queen’s University, Kingston, ON, K7L 3N6, Canada

^2^Interdisciplinary Laboratory of Electrochemistry and Ceramics, Department of Chemistry, Federal University of Sao Carlos, São Carlos, SP, 13565-905, Brazil

^3^Institut National des Sciences Appliquées (I.N.S.A) de Rouen Normandie, 685 Avenue de l'Université, 76800 Saint-Étienne-du-Rouvray, France.

^4^ICFO - Institut de Ciències Fotòniques, The Barcelona Institute of Science and Technology, Barcelona, 08860, Spain.

^*^Corresponding author: [caothang.dinh@queensu.ca](mailto:caothang.dinh@queensu.ca)


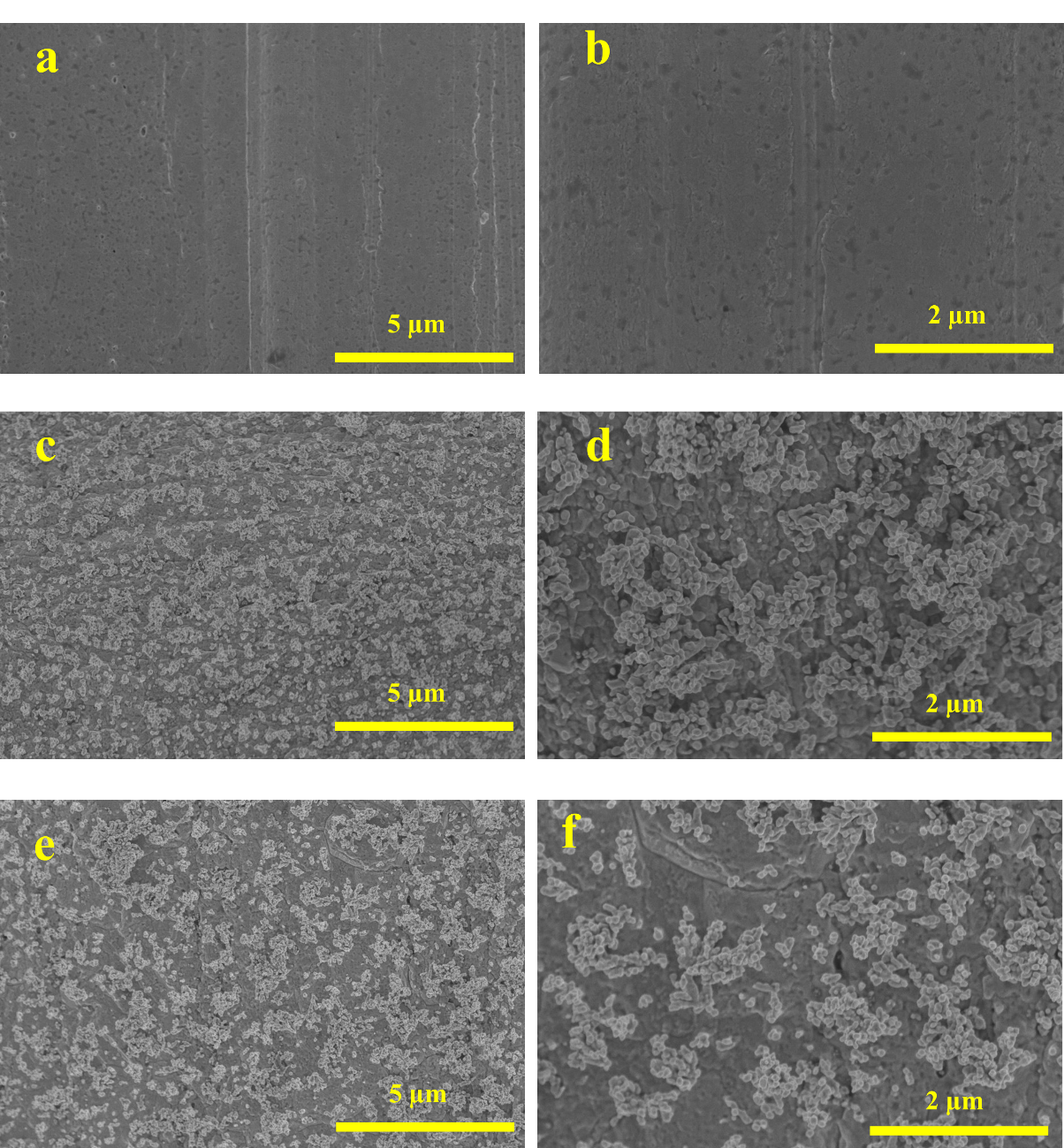


**Figure S1**: The SEM image for Ag mesh at different stages of reaction, (a,b) different magnification of the surface after constant current applying of 100 mA/cm^2^ for 1 hour, (c,d) different magnifications of the surface after pulse condition, and (e,f) different magnification of the surface after constant current third stage of the reaction.


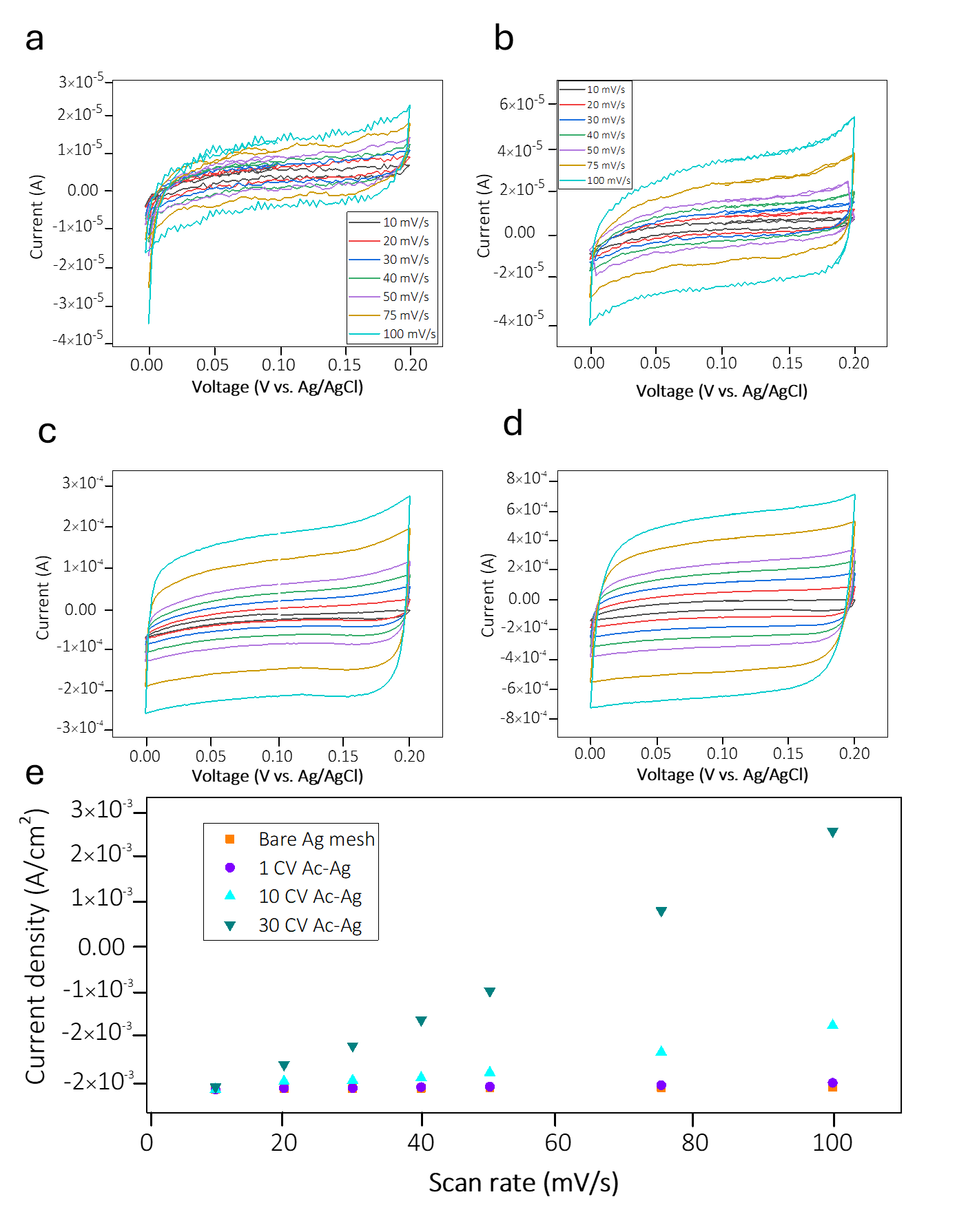


**Figure S2**: (a-d) CVs for calculating electrochemical surface area, (a) bare Ag mesh, (b) 1 CV activated Ag mesh (Ac-Ag), (c) 10 CV Ac-Ag, and (d) 30 CV Ac-Ag, and (e) the scan rate versus current density plot for calculating C_dl_.

**Table S1**: a comparison of the C_dl­_ for different CV treated Ag meshes

| **Catalyst** | **C_dl_ (mF/cm^2^)** | **R^2^** |
| --- | --- | --- |
| Bare Ag mesh | 0.0003 | >99% |
| 1 CV Ac-Ag | 0.0008 | >99% |
| 10 CV Ac-Ag | 0.0076 | >99% |
| 30 CV Ac-Ag | 0.032 | >99% |


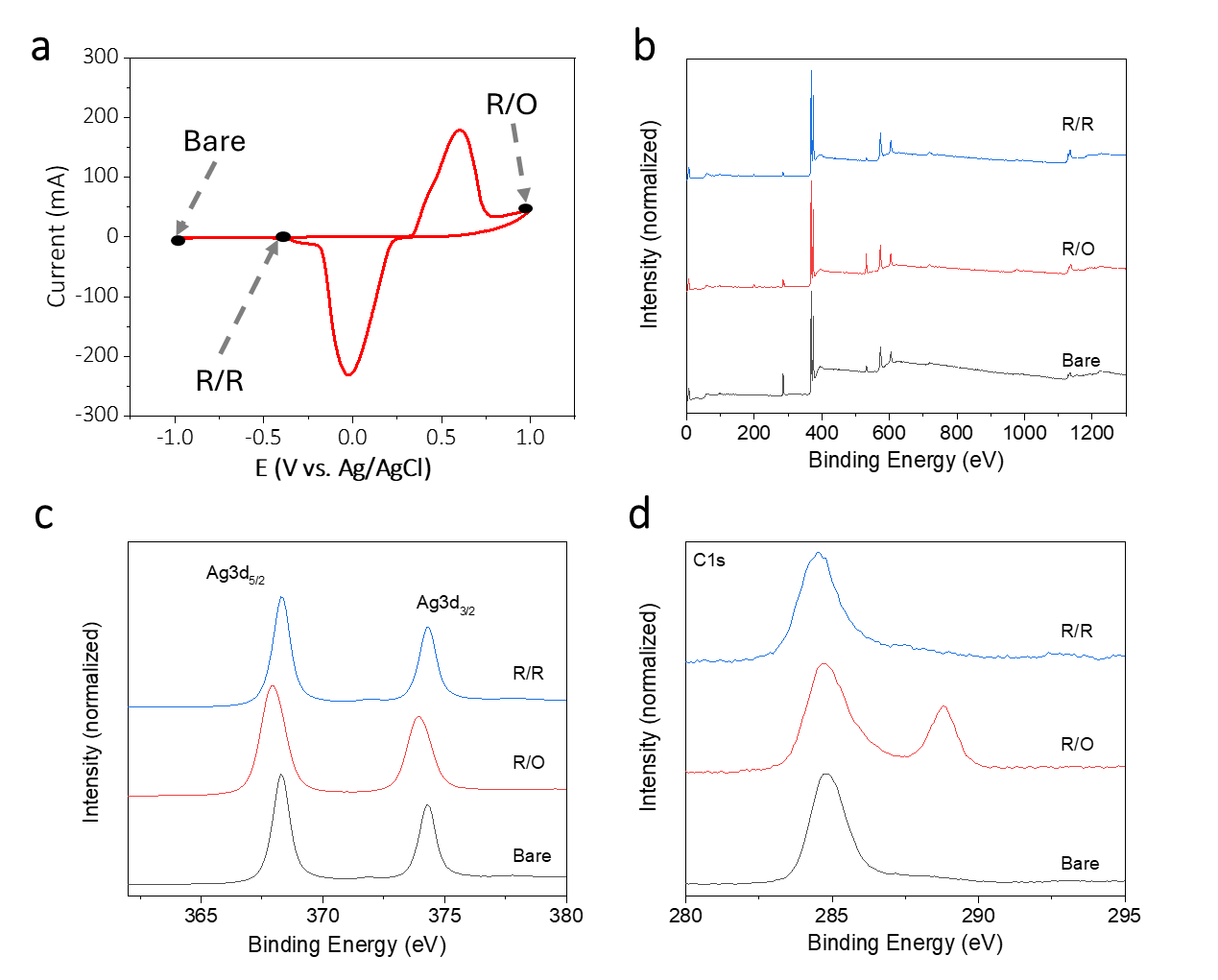


**Figure S3**: (a) details of the conditions that Ag mesh surface was used to analysis, (b) XPS survey spectrum of Ag mesh under three conditions: Bare (no treatment), R/O (oxidized state, stopped at +1V), and R/R (reduced state, stopped at -1V), (c) C1s high-resolution XPS spectra for Bare, R/O, and R/R conditions, (d) C1s high-resolution XPS spectra for Bare, R/O, and R/R conditions.

**
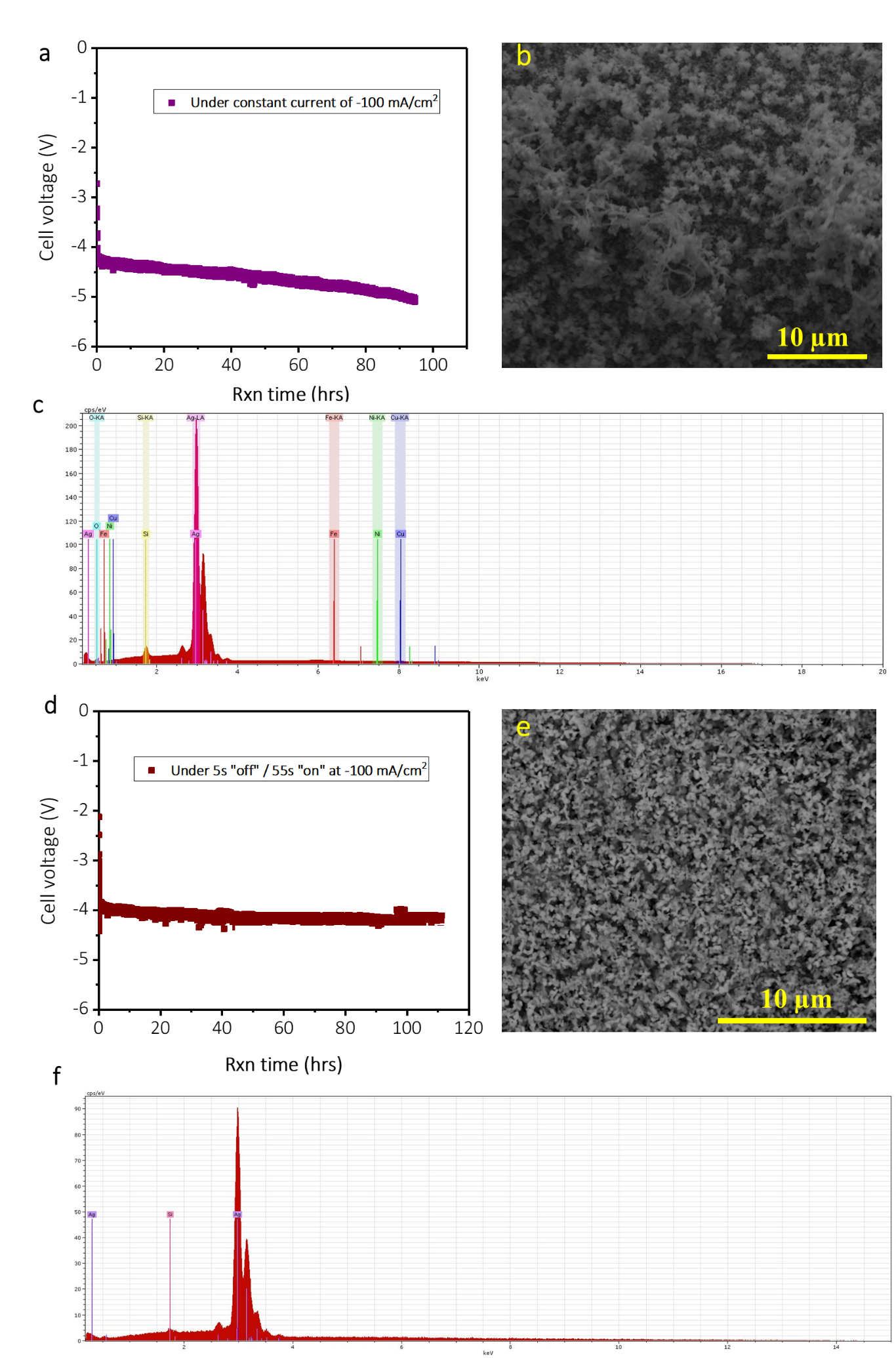
**

**Figure S4**: (a) The cell voltage data over long-time of continuous operation under a constant current density of 100 mA/cm², (b) the SEM image after 100 hours of reaction, (c) EDS spectrum for the Ag surface after long time stability under constant current, (d) The cell voltage vs. reaction time conducted under periodic on/off conditions with a 5s "off" and 55s "on" cycle at a constant current density of 100 mA/cm², (e) the SEM image after on/off stability test, and (f) EDS spectrum for Ag surface after long time stability under “on”/”off” condition.


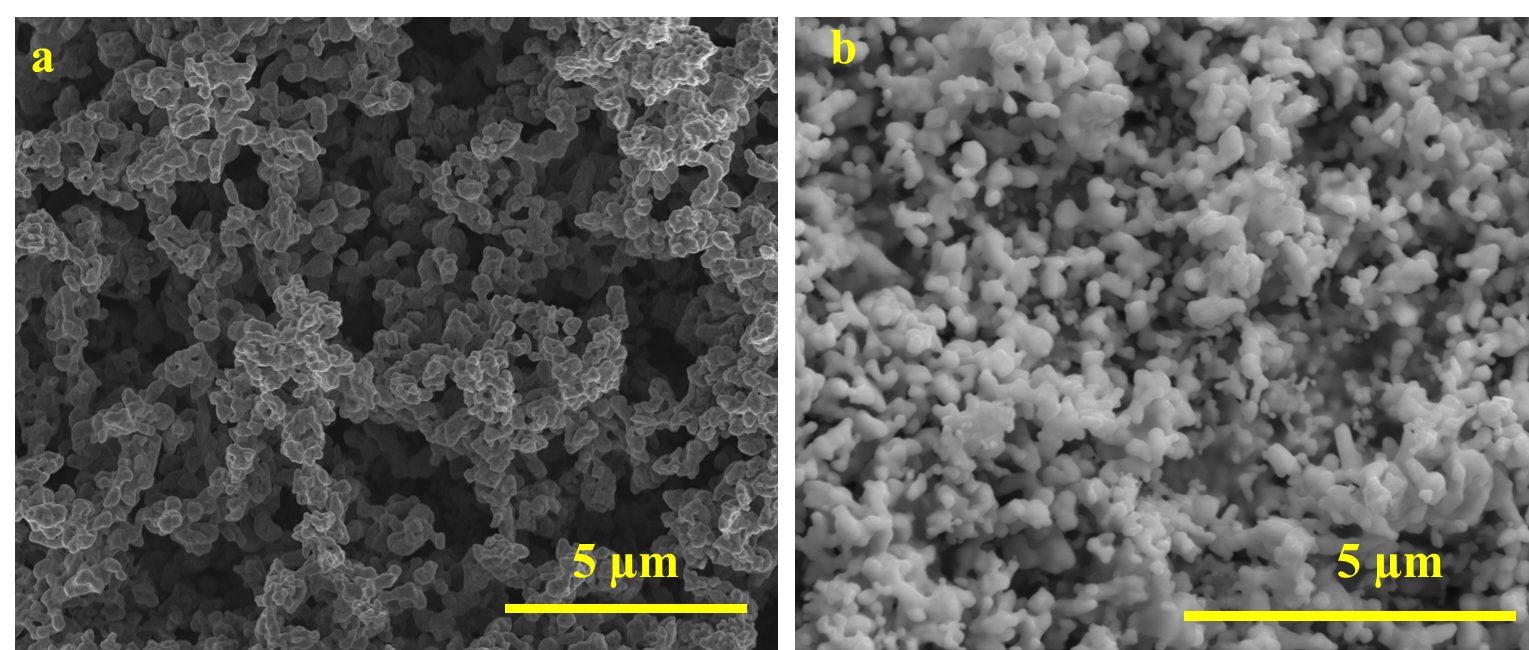

**Figure S5**: (a) SEM image before CO_2_ electroreduction test, and (b) SEM image after long term CO_2_ electroreduction test under “on”/”off” test.

**Figure S6**: the XRD pattern for Ac-Ag catalyst under different conditions; before the CO_2_ER test, and after the long-time stability test under both constant current and on/off conditions.
